# Schizophrenia polygenic risk score in psychosis proneness

**DOI:** 10.1007/s00406-023-01633-7

**Published:** 2023-06-10

**Authors:** Patricia Mas-Bermejo, Sergi Papiol, Marc Via, Paula Rovira, Pilar Torrecilla, Thomas R. Kwapil, Neus Barrantes-Vidal, Araceli Rosa

**Affiliations:** 1 Secció de Zoologia i Antropologia Biològica. Departament de Biologia Evolutiva, Ecologia i Ciències Ambientals. Facultat de Biologia, Universitat de Barcelona, Avda. Diagonal 643, 08028 Barcelona, Spain; 2 Institut de Biomedicina de la Universitat de Barcelona, Barcelona, Spain; 3 Institute of Psychiatric Phenomics and Genomics (IPPG), University Hospital, LMU Munich, 80336 Munich, Germany; 4 Max Planck Institute of Psychiatry, Munich, Germany; 5 CIBER de Salud Mental, Instituto de Salud Carlos III, Madrid, Spain; 6 Brainlab, Cognitive Neuroscience Research Group, Department of Clinical Psychology and Psychobiology, Universitat de Barcelona, Barcelona, Spain; 7 Institut de Neurociències, Universitat de Barcelona, Barcelona, Spain; 8 Institut de Recerca Sant Joan de Déu, Esplugues de Llobregat, Spain; 9 Vicerectorat de Recerca, Investigadora Postdoctoral Margarita Salas, Universitat de Barcelona, Barcelona, Spain; 10 Instituto de Neurociencias, Centro de Investigación Biomédica (CIBM), Universidad de Granada, Granada, Spain; 11 Departamento de Psiquiatría, Facultad de Medicina, Universidad de Granada, Granada, Spain; 12 Instituto de Investigación Biosanitaria Ibs.Granada, Granada, Spain; 13 Department of Clinical and Health Psychology, Universitat Autònoma de Barcelona, Barcelona, Spain; 14 Department of Psychology, University of Illinois at Urbana-Champaign, Champaign, IL USA; 15 Sant Pere Claver-Fundació Sanitària, Barcelona, Spain

**Keywords:** Polygenic risk score, Genetic overlap, Schizophrenia, Schizotypy, Psychotic-like experiences, Motor abnormalities

## Abstract

Schizophrenia (SZ) is a complex disorder with a highly polygenic inheritance. It can be conceived as the extreme expression of a continuum of traits that are present in the general population often broadly referred to as schizotypy. However, it is still poorly understood how these traits overlap genetically with the disorder. We investigated whether polygenic risk for SZ is associated with these disorder-related phenotypes (schizotypy, psychotic-like experiences, and subclinical psychopathology) in a sample of 253 non-clinically identified participants. Polygenic risk scores (PRSs) were constructed based on the latest SZ genome-wide association study using the PRS-CS method. Their association with self-report and interview measures of SZ-related traits was tested. No association with either schizotypy or psychotic-like experiences was found. However, we identified a significant association with the Motor Change subscale of the Comprehensive Assessment of At-Risk Mental States (CAARMS) interview. Our results indicate that the genetic overlap of SZ with schizotypy and psychotic-like experiences is less robust than previously hypothesized. The relationship between high PRS for SZ and motor abnormalities could reflect neurodevelopmental processes associated with psychosis proneness and SZ.

**Supplementary Information:**

The online version contains supplementary material available at 10.1007/s00406-023-01633-7.

## Introduction

Schizophrenia (SZ) is a severe and disabling mental disorder that affects around 24 million people worldwide [1]. Family and twin studies have provided evidence of its multifactorial origin, with a strong genetic component evidenced by heritability estimates around 70–80% [2, 3]. As confirmed over the last few years by genome-wide association studies (GWAS), the genetic architecture of this disorder is highly polygenic, with the cumulative effects of a large number of genes involved. However, although numerous risk loci have already been identified, these variants still only explain a relatively small fraction of the overall heritability of SZ [4, 5].

According to the dimensional view of mental disorders, the psychosis phenotype is manifested across a dynamic continuum in which SZ represents the most extreme of a much broadly distributed clinical expression of psychosis liability expressed as schizotypy traits and psychotic-like experiences in the general population [6–9]. SZ and its subclinical presentations are heterogeneous and this heterogeneity can be captured in a multidimensional structure, with positive, negative and disorganized symptom dimensions most commonly identified. The positive dimension involves odd beliefs ranging from trait-like features such as magical thinking to sub/clinical symptoms like delusions, unusual perceptual experiences that include illusions and hallucinations, suspiciousness and paranoia. The negative or deficit dimension comprises anhedonia, flattened affect, alogia, anergia, and disinterest in the world. And the disorganization dimension involves disruptions in the organization and expression of thought, communication, emotion, and behaviour. These dimensions range from adaptation or minimal dysfunction to overt clinical psychosis, possibly reflecting certain genetic and non-genetic etiological continuity [10–12]. Subclinical traits are hypothesized to be genetically less complex than clinical phenotypes and more directly related to aetiological factors than categorical diagnostic groups, thus being considered interesting candidate phenotypes for the study of SZ [13–16].

To date, little is known about the contribution of genetic risk loci for SZ to SZ-related traits in the general population [17]. In the pre-GWAS era, schizotypy and psychotic-like experiences have been considered as phenotypes in candidate gene studies attempting to identify susceptibility variants related to SZ and both have been found to be associated with previously reported genetic risk variants for this disorder [18–23]. More recently, GWAS have laid the groundwork for the identification of the polygenicity of SZ. One of the tools that GWAS have facilitated is the calculation of Polygenic Risk Scores (PRSs), by computing the sum of an individual’s risk alleles weighted by the effect sizes of such alleles. PRSs provide an estimation of the individual genetic liability to a trait or a disorder and can be used to study the shared genetic aetiology among complex traits at the population level [24].

In the field of psychosis proneness, studies applying PRSs for SZ (SZ-PRSs) to examine the genetic overlap between SZ and its related subclinical phenotypes so far have led to controversial results. On the one hand, several previous studies failed to detect an association between SZ-PRSs and psychotic-like experiences when examining population-based samples of varying sample sizes (e.g., [25–28]). Similarly, Nenadić and colleagues [29] tested the hypothesized association of schizotypy and SZ-PRSs in two non-clinical samples and were not able to find a significant association either. In line with these negative results, a previous study exploring the same hypothesis in a sample of male army recruits reported an inverse association with schizotypy, but follow-up analyses revealed that the association only held under stressful conditions, suggesting an environmental impact rather than a SZ-related genetic influence [30]. However, there is also a growing number of studies with significant findings that support the existence of a shared genetic background between this disorder and its related phenotypes. For instance, four studies examining relatively large general population samples found evidence of an association between SZ-PRSs and multiple measures of psychotic-like experiences [31–34]. Likewise, Karcher and colleagues [35] recently reported an association between SZ-PRSs and distressing psychotic-like experiences in a population-based cohort of children. Regarding schizotypy, Docherty et al. [36] examined a sample of healthy individuals and found a male-specific association between SZ-PRSs and schizotypy. Additionally, van Os et al. [37] reported an association with both positive and negative schizotypy in a similar sample. It should be pointed out that the authors of this last study, as well as Zammit and colleagues [25], used interview-based measures to assess schizotypy and psychotic-like experiences instead of self-report questionnaires, which might have avoided certain phenotypic assessment biases.

Given this background, the aim of this study was to examine the contribution of SZ genetic risk variants to SZ-related traits by analyzing whether SZ-PRSs are associated with SZ-related phenotypes in a sample of non-clinical young adults. The phenotyping of participants was enriched by including the assessment of self-reported traits and psychotic-like experiences as well as face-to-face interviews of a broad range of subclinical experiences and symptoms.

## Sample and methods

### Participants

The sample of the present study was part of the ongoing Barcelona Longitudinal Investigation of Schizotypy Study (BLISS) [38–40]. At T1 of the BLISS, 547 unselected college students enrolled in Psychology courses at the *Universitat Autònoma de Barcelona* (UAB) and 261 students from seven technical training schools in Barcelona were initially screened with self-report questionnaires. At T2, a subsample of 253 individuals (i.e., 214 from the UAB and 39 from technical training schools) oversampled for schizotypy scores to ensure enough variance in the construct of interest was selected to conduct in depth examinations comprising a wide range of interview, questionnaire and experience sampling methodology measurements. This study uses data from the 253 subjects of this T2 subsample, although 25 of them were excluded during the quality control of genetic data. Out of the final 228 participants, 164 were women (71.9%) and 64 were men (28.1%); with a mean age of 19.6 years (SD 2.9, range 17–44) and 20.8 (SD 2.3, range 18–29), respectively. Seven percent of them had a family history of psychotic disorder. All subjects volunteered to take part in the study and provided written informed consent when the assessments were carried. Ethical approval was obtained from local research ethics committees.

### Psychometric assessment

Psychotic-like experiences were assessed with the Spanish version of the Community Assessment of Psychic Experiences (CAPE) [41], which has shown to be valid and reliable in general population samples [42, 43]. This 42-item self-report questionnaire evaluates the lifetime prevalence of three dimensions of symptoms: the positive, negative and depressive dimensions. Frequency is rated on a 4-point Likert scale from 1 (*never*), 2 (*sometimes*), 3 (*often*), to 4 (*nearly always*). The positive and negative dimensions were used in this study.

Schizotypy was assessed with the Wisconsin Schizotypy Scales (WSS), which include the Perceptual Aberration, Magical Ideation, Revised Social Anhedonia and Physical Anhedonia scales [44–47]. Subjects from the technical training schools completed the short version of the self-report scales [48]. Confirmatory factor analyses of the four scales in samples of 6137 and 2292 young adults for the original and short version of the WSS, respectively, revealed a positive and a negative schizotypy factor, which accounted for 80% of the variance [49, 50]. Positive schizotypy tapped magical thinking and abnormal perceptual experiences, whereas negative schizotypy captured social and physical anhedonia. In the present study, participants were assigned positive and negative schizotypy factor scores in order to use them for the analyses, thereby enabling the comparison between data obtained from the short and the original versions of the WSS.

All participants were interviewed by trained psychologists with the Comprehensive Assessment of At-Risk Mental States (CAARMS) [51]. This semi-structured interview consists of seven subscales: (i) Positive symptoms, (ii) Cognitive change, attention, concentration, (iii) Emotional disturbance, (iv) Negative symptoms, (v) Behavioural change, (vi) Motor/physical change and (vii) General psychopathology. Severity ratings were used in this study for each subscale. These variables were dichotomized; splitting individuals who scored zero (no symptoms at all) from those who scored 1 or more (i.e., presenting some degree of symptomatology).

### Genotyping, quality control and imputation

DNA was extracted from saliva or cotton swabs using the prepIT-L2P kit (DNA Genotek Inc., Ottawa, Ontario, Canada) and the RealPure Genomic DNA Extraction Kit (Durviz S.L.U., Valencia, Spain), respectively. DNA samples were genotyped at the *Centro Nacional de Genotipado* (CEGEN-PRB3-ISCIII; CNIO-Madrid) using the Illumina Infinium Global Screening Array-24 v2.0 (GSA) BeadChip. GenomeStudio v2.0.4 (Illumina Inc., San Diego, CA, USA) was used to generate the genotype calls. The quality control (QC) was carried out with PLINK v1.9 (www.cog-genomics.org/plink/1.9/) [52] to exclude SNPs that: had a missing call rate > 2%; had a Minor Allele Frequency (MAF) < 0.1%; or deviated from Hardy–Weinberg equilibrium with a P-value < 0.001. Subjects were excluded when: had a missing call rate > 2%; were related with other participants or duplicated samples according to the pairwise identity by descent method (PI_HAT > 0.25); or had non-European ancestry as inferred with a Multidimensional Scaling (MDS) analysis in which the first 10 ancestry components were extracted. From the total sample of 253 non-clinical individuals, 25 subjects were excluded due to this exhaustive QC leaving a sample of 228 subjects. MDS components were then recalculated in this final sample and the first two were used in all models as independent variables. Imputation was carried out using the Haplotype Reference Consortium panel (www.haplotype-reference-consortium.org) [53] in the Michigan Imputation Server [54]. Post-imputation QC was performed to filter out SNPs with a MAF < 1% and Rsq < 0.3. A total of 7,755,414 SNPs passed post-imputation QC.

### Calculation of PRSs

PRSs were calculated for each of the 228 participants based on the latest SZ GWAS of the Psychiatric Genomics Consortium [4]. PRS-CS tool was used to infer posterior SNP effect sizes under continuous shrinkage priors [55]. We did a small-scale grid search setting the global shrinkage parameter *phi* (φ) at: 1.00E−01, 1.00E−02, 1.00E−03, 1.00E−04, 1.00E−05 and 1.00E−06 besides selecting the auto setting, where φ is automatically learnt using a fully Bayesian approach. The polygenic profiles were computed by summing the number of risk alleles that each individual carries multiplied by the inferred posterior SNP weights, including a total of 1,107,471 SNPs.

### Statistical analyses

All analyses were conducted using RStudio (v1.1.456; RStudio, Inc.). An a priori power analysis was carried out with the pwr.f2.test function of the pwr R package (v1.3.0) in order to estimate the minimum effect size that we could identify with a power of 80% considering our current sample size (N = 228) and an alpha level of 0.05. Result showed that the minimum effect size detectable was *f*^2^ = 0.064 (i.e., *R*^2^ = 0.061). The association between the psychometric variables and the polygenic scores at the 7 different φ values was tested using linear and logistic regression models for the continuous and dichotomized variables, respectively. The analyses were performed for both the total sample and by sex, and were adjusted for age, sex, recruitment center (i.e., UAB or technical training schools students) and the first two ancestry-based MDS components (excluding sex in sex-stratified analyses). The amount of variance on the psychometric variables explained by the PRSs alone was estimated calculating the incremental Adjusted *R*^2^ (incr. Adj. *R*^2^) for continuous variables and the incremental Nagelkerke’s pseudo-*R*^2^ (incr. Nagelkerke’s *R*^2^) for dichotomized variables, which are the difference in Adjusted *R*^2^ or Nagelkerke’s *R*^2^ between the full model and the baseline model (i.e., including all variables except the PRSs). We applied the False Discovery Rate (FDR) method [56] correcting for the PRSs derived from the 7 different φ values to correct for multiple testing.

## Results

### Association between PRSs for SZ and the self-report scales

Of the 228 non-clinical subjects with genetic data, 226 had valid scores for the CAPE (i.e., assessment of psychotic-like experiences) and all 228 participants completed the WSS (i.e., schizotypy assessment). Descriptive statistics and correlations between the two measures are presented in Table 1.

**Table 1:** Descriptive statistics and Spearman’s correlations of the self-report scales

		Descriptive statistics		Spearman’s correlations				
		Mean (SD)	Range	CAPE dimension		WSS factor		
				Positive	Negative	Positive		Negative
CAPE dimension	Positive	8.48 (5.04)	0 to 23		0.382***	0.697***		0.125
	Negative	10.31 (5.62)	0 to 35			0.377***		0.436***
WSS factor	Positive	− 0.32 (0.85)	− 1.56 to 2.24					0.115
	Negative	0.01 (1.03)	− 1.57 to 4.27					

*SD* standard deviation

***Correlation is significant at *P* < 0.001

Linear regression analyses performed to test the association between SZ-PRSs and the scores of the two dimensions of psychotic-like experiences showed no significant results for any of the 7 φ values tested (Supplementary Table S1). Likewise, no associations were found between the positive and negative schizotypy scores and the SZ-PRSs derived from the 7 φ values (Supplementary Table S2).

### Association between SZ-PRSs and the CAARMS interview subscales

The CAARMS subscales were available for all 228 participants, with the exception of the General Psychopathology subscale, for which data was available for 218 individuals. The frequency distribution of the dichotomized seven subscales is reported in Table 2.

**Table 2:** Frequency distribution of the CAARMS interview subscales

CAARMS subscale		Frequency	Valid percent
Positive symptoms	0	141	61.8
	$\ge$ 1	87	38.2
Cognitive change	0	138	60.5
	$\ge$ 1	90	39.5
Emotional disturbance	0	158	69.3
	$\ge$ 1	70	30.7
Negative symptoms	0	131	57.5
	$\ge$ 1	97	42.5
Behavioural change	0	130	57
	$\ge$ 1	98	43
Motor change	0	142	62.3
	$\ge$ 1	86	37.7
General psychopathology	0	66	30.3
	$\ge$ 1	152	69.7

Logistic regressions testing the association between the SZ-PRSs and the seven subscales of the CAARMS showed a significant association of the Motor Change subscale with the PRSs derived from 5 of the 7 φ values tested (Table 3). This association survived FDR correction. The maximum amount of variance on this variable was explained by the PRS computed at φ = 1.00E−02 (incr. Nagelkerke’s *R*^2^ = 5.2%), which showed an odds ratio (OR) of 2.56 indicating that in our sample it is 2.56 times more likely to present motor changes for each increase of one standard deviation of the PRS.

**Table 3:** Results of the regression models between the CAARMS Motor Change subscale and the PRSs derived from the 7 φ values of the PRS-CS method

φ value	Effect (β)	Nagelkerke’s *R*^2^ model	Incr. Nagelkerke’s *R*^*2*^	OR	*P* value	FDR* P* value
1.00E−01	0.657	0.102	0.050	1.92	0.003**	0.012*
1.00E−02	0.940	0.104	0.052	2.56	0.003**	0.012*
1.00E−03	1.266	0.096	0.044	3.54	0.006**	0.015*
1.00E−04	1.512	0.082	0.030	4.53	0.024*	0.034*
1.00E−05	1.493	0.069	0.016	4.45	0.095	0.111
1.00E−06	1.309	0.062	0.009	3.70	0.204	0.204
Auto	1.656	0.093	0.040	5.23	0.009**	0.016*

*OR* Odds Ratio

*Association is significant at *P* < 0.05; **association is significant at *P* < 0.01

We did not find significant associations between the other CAARMS subscales and any of the φ values tested (Supplementary Table S3).

**Fig. 1:**
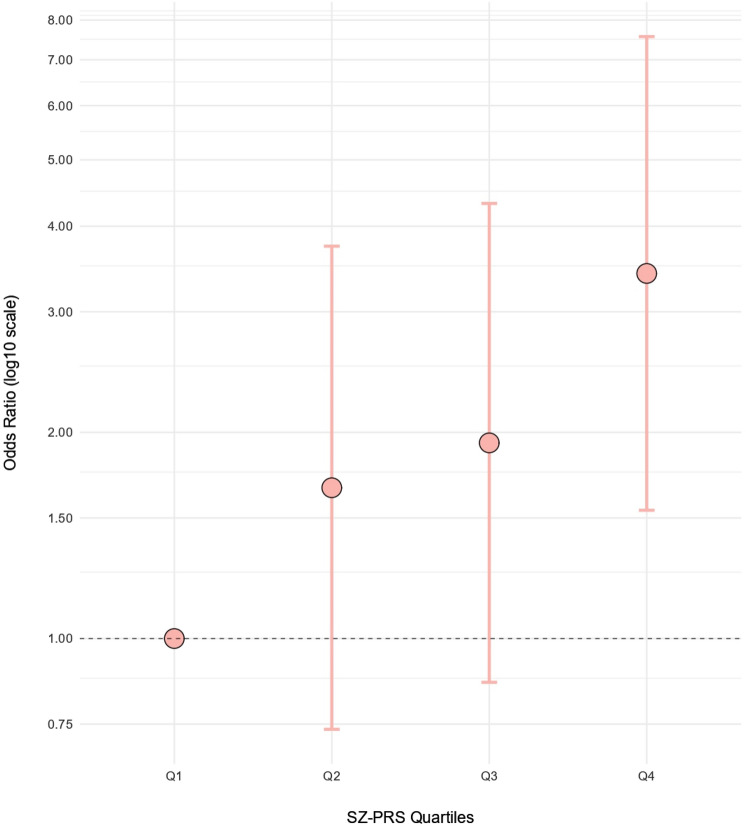
Motor change risk increase with the polygenic burden of SZ. Odds ratios (OR) for the risk of presenting CAARMS Motor Change score > 0 between high and low SZ-PRS after dividing the sample based on quartiles using the standard residuals of the SZ-PRS corrected for age, sex, recruitment center, and the first two ancestry-based MDS components

To better visualize the effect of the SZ-PRSs on the scores of the Motor Change subscale, we constructed a plot representing the variation of the ORs for the risk of scoring > 0 on this subscale with increasing SZ-PRSs (Fig. 1). The sample was split based on quartiles using the standard residuals of the SZ-PRSs derived from the auto setting and corrected for age, sex, recruitment center, and the first two ancestry-based MDS components. Significant differences were found between the lowest and highest strata of data (Q1 and Q4, respectively; OR 3.41; *P* = 0.003) pointing to an increasing risk of motor changes with increasing PRS values.

### Association of SZ-PRSs and the psychometric variables by sex

Sex-stratified analyses revealed a significant association of the SZ-PRSs with the CAARMS Motor Change subscale in women, for 4 of the 7 φ values tested. The PRS computed at φ = 1.00E−01 explained the maximum amount of variance on this variable (incr. Nagelkerke’s *R*^2^ = 5%; OR 1.96). However, these results did not survive FDR correction (Supplementary Table S4). No significant associations were found when performing the analyses in men (Supplementary Table S5).

## Discussion

The present study aimed to investigate in a non-clinically ascertained sample whether SZ-PRSs were associated with SZ-related subclinical phenotypes, that is, schizotypy dimensions, psychotic-like experiences and interview-ratings of a broad range of subclinical experiences and symptoms.

When analyzing the two self-report assessments, we did not find any significant association of the polygenic burden for SZ with psychotic-like experiences or with schizotypy. In the past years, in accordance with an increasing support for the psychosis extended phenotype hypothesis (e.g., [9–11]), many attempts have been made in order to find evidence of an overlapping genetic architecture between SZ and its related phenotypes. However, the previous literature specifically examining psychotic-like experiences and schizotypy in relation to SZ-PRSs shows inconsistent results, so the existence of a genetic overlap with these phenotypes is still unclear. Although some studies have found evidence of a shared genetic aetiology with different measures of psychotic-like experiences and schizotypy [31–34, 36], our findings indicating a lack of an association concurs with several other studies. For instance, both Sieradzka et al. [26] and Zammit et al. [25] examined in relatively large population-based samples whether SZ-PRSs were associated with different measures of psychotic-like experiences and neither of them identified any significant association. In line with these negative results, van Os et al. [28] investigated in two independent healthy comparison samples whether SZ-PRSs were associated with psychotic-like experiences assessed with the CAPE self-report questionnaire and did not detect any significant association either. Finally, our results are consistent with those of Nenadić and colleagues [29], who were unable to identify any significant association when exploring the genetic overlap between SZ-PRSs and schizotypy in two non-clinical samples. This led them to propose that schizotypy should be regarded as a wider phenotype beyond merely harbouring risk for SZ, consistent with dimensional conceptualizations of the dual nature of schizotypy, as any other trait, as an indicator of both normal individual differences as well as behavioural risk for psychosis [57]. Therefore, specific genes giving risk for SZ may in a sense wash out in the larger pool of schizotypic individuals, which does not diminish the utility of schizotypy, but highlights that it is an interesting construct in its own right, not simply a prodromal or risk condition for SZ. Nevertheless, the comparison of previous results between these types of studies should be done with caution as the different instruments and questionnaires used for the psychometric assessment could be capturing different underlying concepts [58].

Other plausible explanations for such a lack of associations in the literature have been pointed out. Nenadić et al. [29] suggested the possibility that, in the non-clinical part of the psychosis continuum, environmental stressors may have a larger effect on the phenotypic expression of SZ-related traits than genetic predisposition, which would be in consonance with the low variance of different subclinical phenotypes that SZ-PRSs explain. Some studies have already found evidence of an environmental contribution to the expression of psychotic-like experiences and schizotypy in samples from the general population. For example, a link between psychotic-like experiences and smoking and using cannabis in general population samples has been described [59–61]. In a similar fashion, Pries and colleagues [62] computed a score of cumulative environmental load that included childhood adversity, winter-birth, cannabis use, and hearing impairment and found that it was associated with positive, negative, and total schizotypy. Additionally, in line with these findings a previous work identified an association between SZ genetic load and positive schizotypy in a sample of male army recruits, but only at the stressed condition of military induction, which denoted an environmental influence [30]. It is also likely that in the non-clinical end of the psychosis continuum, plasticity alleles rather than risk alleles (i.e., alleles that confer sensitivity to both positive and negative environmental influences rather than alleles that only confer vulnerability in the presence of environmental adversity) play a more relevant role in the underlying pathways that lead to the expression (or not) of these subclinical manifestations. Therefore, PRSs that likely reflect susceptibility to environmental influences rather than risk to develop SZ might better capture the genetic architecture of these subclinical traits [63].

Another plausible explanation for the increasing number of negative results in previous research could be that, like SZ, its related subclinical phenotypes might also be determined by different types of genetic variants beyond common SNPs considered in PRS computation. In fact, SNP-based heritability estimates for SZ indicate that common variation only explains around 24% of the variance in SZ liability [4]. Therefore, the lack of significant associations cannot entirely rule out the possibility of a genetic overlap between these phenotypes, as it could mainly be conformed of other types of genetic variation such as copy number variants or rare variants. Finally, it could be that the PRSs constructed based on variants associated with clinically diagnosed SZ might not be able to capture the subclinical manifestations of psychosis since these typically overlap within a transdiagnostic mix of symptoms [64]. Thus, future research using PRSs built with variants associated with these subclinical symptoms in non-clinical or at-risk samples could elucidate this question.

Regarding the CAARMS interview, our findings seem to identify an association between the polygenic risk for SZ and the presence of motor abnormalities. We found an association with the Motor/physical Change subscale, which evaluates subjectively experienced difficulties with movement and objective signs of catatonia, including: subjective complaints of impaired motor functioning; informant reported or observed changes in motor functioning; subjective complaints of impaired bodily sensation; and subjective complaints of impaired autonomic functioning [51]. Individuals reporting some degree of motor abnormalities on the Motor Change subscale presented higher SZ-PRSs, which suggests that non-clinical individuals with a higher end of polygenic burden for SZ already present some degree of motor dysfunction despite being functional young adults, in comparison with those subjects with a low SZ polygenic load. In fact, when we divided our sample into quartiles of increasing PRS, a trend towards an increase in motor changes with increasing SZ polygenic load could be observed, where the risk of presenting some motor abnormalities was three times higher for individuals in the highest quartile than those in the lowest quartile. Given that SZ is known to affect men and women differently [65], the association analyses were also conducted based on gender. The association between the SZ polygenic burden and the presence of motor abnormalities found in the whole sample was also detected in the female subsample, although in this case all significance was lost after FDR correction. Regarding men, no significant association was found. However, given that the size of the male subsample was considerably small (N = 64), we cannot rule out the possibility that this association also exists in men. Moreover, it could be observed that the association was stronger for the whole sample than for the female subset only, which suggests that men were actually contributing to the significance of the association, rather than diminishing it. Nevertheless, this should be considered cautiously, given that we did not have specific hypotheses about this domain relative to the other domains tapped by the CAARMS.

Some studies have estimated that up to 80% of patients with SZ present some motor anomalies, already observed very early during premorbid development in most of these patients [66]. These abnormalities have been associated with poorer psychopathological, cognitive, and social outcomes [67–69]. In fact, motor impairment constitutes a key transdiagnostic feature indexing disease severity [70, 71] and a risk factor for conversion to psychosis [72, 73]. Consistent with recent claims of a severity continuum of psychopathology, in which established psychosis (e.g., SZ) might index the extreme end of this continuum [74], SZ-PRSs might also be reflective of a severity score and thus, be more likely to detect most severe manifestations in a non-clinical sample of young adults. The association between SZ-PRSs and the presence of motor abnormalities might be detecting those individuals with poorer functioning and with increased liability for transitioning to clinical at-risk states. It remains to be established how these results connect with neurodevelopmental processes and if PRSs are able to finally shed a light in the interplay between genetic risk, neurodevelopmental processes, and subclinical traits in the general population.

The findings of the present study should be interpreted with caution bearing in mind some limitations. On the one hand, given the face-to-face nature of part of the psychometric assessment, our sample was relatively small for the type of investigation carried out and a slight lack of statistical power has to be acknowledged. On the other hand, the sex-stratified analyses performed may be biased since more than 70% of the participants were women and the size of the male subsample (N = 64) will likely have affected our statistical power. Additionally, we had no knowledge of the developmental and drug use history of the participants, which could have added an interesting insight to the analyses carried out. Finally, due to the non-clinical nature of the sample analyzed in this study, most participants reported relatively low scores on the psychometric scales. On the CAARMS interview, especially, a clear floor effect could be observed as this interview was initially designed to evaluate help-seeking individuals rather than non-clinical subjects—although we highlight that the present sample was oversampled for both positive and negative schizotypy and psychotic-like experiences from a larger unselected sample. Thus, the CAARMS variables were dichotomized, which might have led to a loss of statistical power [75]. Even so, it is worth noting that we were still able to detect a robust association with the Motor Change subscale of this interview.

## Conclusions

In conclusion, our results seem to suggest that schizotypy and psychotic-like experiences share less genetic variability with SZ than initially hypothesized and that SZ-PRSs are able to capture the subjective motor changes measured with the CAARMS interview presented in some healthy subjects from the general population. Further studies in larger non-clinical samples are required in order to finally unravel the shared genetic background between this disorder and its subclinical phenotypes and to ensure the replicability of the association with motor abnormalities found in the present study. Moreover, the replication of this finding across levels of severity expression (e.g., at risk mental states and first episode psychosis) would allow us to both understand more in depth the nature of this association and test whether, as it would be expected, this association becomes more evident at greater severity expressions of the extended psychosis phenotype that presumably index increasing genetic load for SZ.

### Supplementary Information

Below is the link to the electronic supplementary material.

Supplementary file1 (DOCX 34 KB)

## Data Availability

The data that support the findings of this study are available from the authors AR and NB-V upon reasonable request.
