# Supplementary material for: Schizophrenia polygenic risk score in psychosis proneness
Source: Eur Arch Psychiatry Clin Neurosci. 2023 Jun 10;273(8):1665–75. doi: 10.1007/s00406-023-01633-7 (PMC10713704; doi:10.1007/s00406-023-01633-7)
Supplement: Supplementary file 1 — Supplementary file1 (DOCX 34 KB) [file 406_2023_1633_MOESM1_ESM.docx]

**Supplementary Information for**

**Article Title**: Schizophrenia polygenic risk score in psychosis proneness

**Journal name**: European Archives of Psychiatry and Clinical Neuroscience

**Authors**: Patricia Mas-Bermejo 1,2,‡, Sergi Papiol 3,4,5,‡, Marc Via 6,7,8, Paula Rovira 9,10,11,12, Pilar Torrecilla 13, Thomas R Kwapil 14, Neus Barrantes-Vidal 5,13,15, Araceli Rosa 1,2,5,*

‡ Both authors contributed equally to this work.

1 Secció de Zoologia i Antropologia Biològica, Departament de Biologia Evolutiva, Ecologia i Ciències Ambientals, Universitat de Barcelona, Barcelona, Spain;

2 Institut de Biomedicina de la Universitat de Barcelona, Barcelona, Spain;

3 Institute of Psychiatric Phenomics and Genomics (IPPG), University Hospital, LMU Munich, Munich, 80336, Germany;

4 Max Planck Institute of Psychiatry, Munich, Germany;

5 CIBER de Salud Mental, Instituto de Salud Carlos III, Madrid, Spain.

6 Brainlab, Cognitive Neuroscience Research Group, Department of Clinical Psychology and Psychobiology, Universitat de Barcelona, Barcelona, Spain;

7 Institut de Neurociències, Universitat de Barcelona, Barcelona, Spain;

8 Institut de Recerca Sant Joan de Déu, Esplugues de Llobregat, Spain;

9 Vicerectorat de Recerca, Investigadora postdoctoral Margarita Salas, Universitat de Barcelona, Barcelona, Spain;

10 Instituto de Neurociencias, Centro de Investigación Biomédica (CIBM), Universidad de Granada, Granada, Spain;

11 Departamento de Psiquiatría, Facultad de Medicina, Universidad de Granada, Granada, Spain;

12 Instituto de Investigación Biosanitaria ibs.Granada, Granada, Spain;

13 Department of Clinical and Health Psychology, Universitat Autònoma de Barcelona, Barcelona, Spain;

14 Department of Psychology, University of Illinois at Urbana-Champaign, Champaign, Illinois, United States of America;

15 Sant Pere Claver- Fundació Sanitària, Barcelona, Spain;

* Corresponding author at: Secció de Zoologia i Antropologia Biològica. Departament de Biologia Evolutiva, Ecologia i Ciències Ambientals. Facultat de Biologia. Universitat de Barcelona, Avda. Diagonal 643, 08028, Barcelona, Spain. E-mail address: [araceli.rosa@ub.edu](mailto:araceli.rosa@ub.edu)

**Supplementary Table S1.** *Results of the regression models between the CAPE dimensions and the SZ-PRSs*

| CAPE dimension | φ value | Effect (β) | Adj. R^2^ model | Incr. Adj. R^2^ | *P*-value |
| --- | --- | --- | --- | --- | --- |
| Positive | 1.00E-01 | 0.109 | 0.006 | -0.004 | 0.833 |
|  | 1.00E-02 | 0.203 | 0.006 | -0.004 | 0.778 |
|  | 1.00E-03 | 0.167 | 0.006 | -0.004 | 0.874 |
|  | 1.00E-04 | 0.141 | 0.006 | -0.004 | 0.927 |
|  | 1.00E-05 | 1.082 | 0.007 | -0.003 | 0.596 |
|  | 1.00E-06 | 0.601 | 0.006 | -0.004 | 0.801 |
|  | auto | 0.443 | 0.006 | -0.004 | 0.756 |
| Negative | 1.00E-01 | 0.342 | -0.001 | -0.003 | 0.555 |
|  | 1.00E-02 | 0.554 | -0.001 | -0.002 | 0.493 |
|  | 1.00E-03 | 1.051 | 0.001 | -0.001 | 0.373 |
|  | 1.00E-04 | 1.456 | 0.001 | -0.001 | 0.395 |
|  | 1.00E-05 | 2.897 | 0.005 | 0.003 | 0.205 |
|  | 1.00E-06 | 3.294 | 0.004 | 0.002 | 0.217 |
|  | auto | 1.367 | 0.001 | -0.001 | 0.392 |

**Supplementary Table S2.** *Results of the regression models between positive and negative schizotypy and the SZ-PRSs*

| WSS factor | φ value | Effect (β) | Adj. R^2^ model | Incr. Adj. R^2^ | *P*-value |
| --- | --- | --- | --- | --- | --- |
| Positive | 1.00E-01 | 0.119 | 0.027 | 0.004 | 0.170 |
|  | 1.00E-02 | 0.164 | 0.027 | 0.004 | 0.174 |
|  | 1.00E-03 | 0.246 | 0.027 | 0.004 | 0.162 |
|  | 1.00E-04 | 0.288 | 0.024 | 0.001 | 0.259 |
|  | 1.00E-05 | 0.444 | 0.026 | 0.003 | 0.193 |
|  | 1.00E-06 | 0.411 | 0.023 | 0.000 | 0.303 |
|  | auto | 0.271 | 0.024 | 0.001 | 0.254 |
| Negative | 1.00E-01 | -0.051 | 0.082 | -0.003 | 0.613 |
|  | 1.00E-02 | -0.075 | 0.083 | -0.003 | 0.593 |
|  | 1.00E-03 | -0.101 | 0.082 | -0.003 | 0.624 |
|  | 1.00E-04 | -0.192 | 0.083 | -0.002 | 0.520 |
|  | 1.00E-05 | -0.214 | 0.083 | -0.003 | 0.592 |
|  | 1.00E-06 | -0.298 | 0.083 | -0.002 | 0.522 |
|  | auto | -0.195 | 0.083 | -0.002 | 0.483 |

**Supplementary Table S3.** *Non-significant results of the regression models between the CAARMS interview subscales and the SZ-PRSs*

| CAARMS subscale | φ value | Effect (β) | Nagelkerke's R^2^ model | Incr. Nagelkerke's R^2^ | *P*-value |
| --- | --- | --- | --- | --- | --- |
| Positive symptoms | 1.00E-01 | 0.187 | 0.127 | 0.004 | 0.399 |
|  | 1.00E-02 | 0.231 | 0.126 | 0.003 | 0.455 |
|  | 1.00E-03 | 0.333 | 0.126 | 0.003 | 0.461 |
|  | 1.00E-04 | 0.566 | 0.127 | 0.004 | 0.390 |
|  | 1.00E-05 | 0.824 | 0.128 | 0.005 | 0.352 |
|  | 1.00E-06 | 0.948 | 0.128 | 0.005 | 0.361 |
|  | auto | 0.497 | 0.127 | 0.004 | 0.417 |
| Cognitive Change | 1.00E-01 | 0.066 | 0.078 | 0.001 | 0.758 |
|  | 1.00E-02 | 0.045 | 0.077 | 0.000 | 0.880 |
|  | 1.00E-03 | 0.180 | 0.078 | 0.001 | 0.682 |
|  | 1.00E-04 | 0.501 | 0.081 | 0.003 | 0.436 |
|  | 1.00E-05 | 0.886 | 0.083 | 0.006 | 0.307 |
|  | 1.00E-06 | 0.918 | 0.082 | 0.005 | 0.365 |
|  | auto | 0.454 | 0.080 | 0.003 | 0.447 |
| Emotional Disturbance | 1.00E-01 | 0.044 | 0.115 | 0.000 | 0.848 |
|  | 1.00E-02 | 0.086 | 0.115 | 0.000 | 0.790 |
|  | 1.00E-03 | 0.114 | 0.115 | 0.000 | 0.809 |
|  | 1.00E-04 | 0.087 | 0.114 | 0.000 | 0.899 |
|  | 1.00E-05 | -0.187 | 0.115 | 0.000 | 0.836 |
|  | 1.00E-06 | -0.724 | 0.117 | 0.003 | 0.492 |
|  | auto | 0.178 | 0.115 | 0.000 | 0.781 |
| Negative symptoms | 1.00E-01 | 0.380 | 0.118 | 0.017 | 0.082 |
|  | 1.00E-02 | 0.478 | 0.115 | 0.014 | 0.116 |
|  | 1.00E-03 | 0.644 | 0.113 | 0.012 | 0.148 |
|  | 1.00E-04 | 0.946 | 0.113 | 0.012 | 0.146 |
|  | 1.00E-05 | 1.234 | 0.112 | 0.011 | 0.159 |
|  | 1.00E-06 | 1.685 | 0.116 | 0.015 | 0.104 |
|  | auto | 0.889 | 0.113 | 0.012 | 0.142 |
| Behavioural Change | 1.00E-01 | 0.183 | 0.153 | 0.004 | 0.402 |
|  | 1.00E-02 | 0.253 | 0.153 | 0.004 | 0.407 |
|  | 1.00E-03 | 0.525 | 0.157 | 0.007 | 0.242 |
|  | 1.00E-04 | 0.588 | 0.154 | 0.004 | 0.366 |
|  | 1.00E-05 | 0.836 | 0.154 | 0.005 | 0.337 |
|  | 1.00E-06 | 0.973 | 0.154 | 0.005 | 0.340 |
|  | auto | 0.761 | 0.158 | 0.008 | 0.213 |
| General Psychopathology | 1.00E-01 | 0.478 | 0.106 | 0.024 | 0.052 |
|  | 1.00E-02 | 0.598 | 0.102 | 0.020 | 0.076 |
|  | 1.00E-03 | 0.943 | 0.105 | 0.023 | 0.057 |
|  | 1.00E-04 | 1.250 | 0.102 | 0.020 | 0.075 |
|  | 1.00E-05 | 1.752 | 0.104 | 0.022 | 0.061 |
|  | 1.00E-06 | 1.463 | 0.093 | 0.011 | 0.171 |
|  | auto | 1.146 | 0.101 | 0.019 | 0.081 |

**Supplementary Table S4.** *Results of the regression models between the SZ-PRSs and the Motor Change subscale of the CAARMS performed in women*

| φ value | Effect (β) | Nagelkerke’s *R^2^* model | Incr. Nagelkerke’s *R^2^* | OR | *P*-value | FDR *P*-value |
| --- | --- | --- | --- | --- | --- | --- |
| 1.00E-01 | 0.673 | 0.075 | 0.050 | 1.96 | 0.013* | 0.055 |
| 1.00E-02 | 0.927 | 0.073 | 0.049 | 2.52 | 0.015* | 0.055 |
| 1.00E-03 | 1.216 | 0.064 | 0.040 | 3.37 | 0.029* | 0.055 |
| 1.00E-04 | 1.466 | 0.051 | 0.027 | 4.33 | 0.072 | 0.102 |
| 1.00E-05 | 1.698 | 0.044 | 0.020 | 5.46 | 0.122 | 0.142 |
| 1.00E-06 | 1.378 | 0.034 | 0.010 | 3.96 | 0.271 | 0.271 |
| auto | 1.635 | 0.063 | 0.039 | 5.13 | 0.031* | 0.055 |

OR, Odds Ratio. *, association is significant at *P* <.05.

**Supplementary Table S5.** *Results of the regression models between the SZ-PRSs and the Motor Change subscale of the CAARMS performed in men*

| φ value | Effect (β) | Nagelkerke’s *R^2^* model | Incr. Nagelkerke’s *R^2^* | *P*-value |
| --- | --- | --- | --- | --- |
| 1.00E-01 | 0.271 | 0.290 | 0.006 | 0.536 |
| 1.00E-02 | 0.567 | 0.298 | 0.014 | 0.351 |
| 1.00E-03 | 0.799 | 0.297 | 0.013 | 0.376 |
| 1.00E-04 | 0.983 | 0.294 | 0.010 | 0.438 |
| 1.00E-05 | 0.365 | 0.284 | 0.0008 | 0.823 |
| 1.00E-06 | 0.305 | 0.284 | 0.0004 | 0.876 |
| auto | 1.028 | 0.296 | 0.012 | 0.392 |
